# Supplementary material for: DeepSlice: rapid fully automatic registration of mouse brain imaging to a volumetric atlas
Source: Nat Commun. 2023 Sep 21;14:5884. doi: 10.1038/s41467-023-41645-4 (PMC10514056; doi:10.1038/s41467-023-41645-4)
Supplement: Supplementary file 1 — Supplementary Information [file 41467_2023_41645_MOESM1_ESM.pdf]

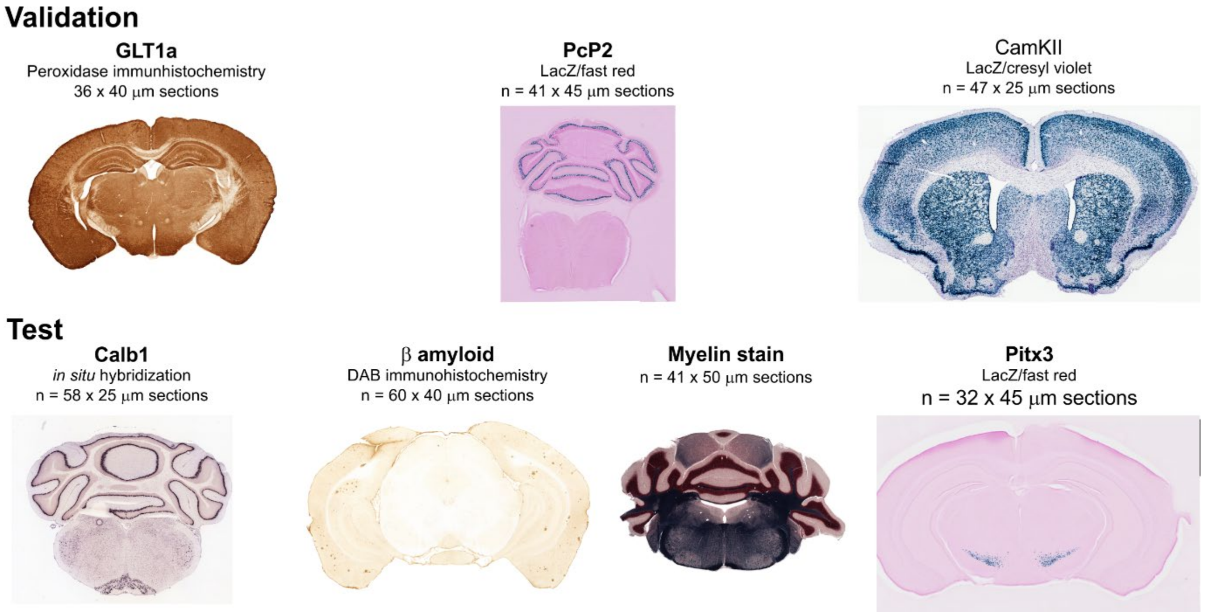

Supplementary Figure 1: Samples of slide-mounted histology used for generation of Validation and Test datasets.

Each dataset was independently aligned by seven human operators; DeepSlice performance was used to guide model development (Validation) and assessment of the final model (Test). Further details of experimental preparation are provided in Table 1. Abbreviations: GLT1a: glutamate transporter 1; PcP2: Purkinje Cell Protein 2; CamKII: Calcium/calmodulin-dependent protein kinase II; Calb1: Calbindin1, DAB: 3, 3'-diaminobenzidine; Pitx3: Paired Like Homeodomain 3. Experiment details provided in Table 1.

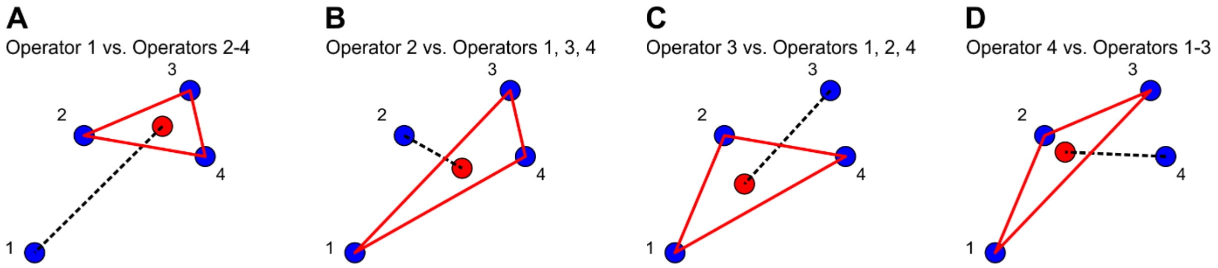

Supplementary Figure 2: Approach used for determination of Ground Truth.

The accuracy of anchoring coordinates generated by each operator (blue circles) was quantified by measuring the distance between each coordinate and a point representing the average of coordinates produced by the other operators (red circle). In this simplified schematic diagram, each panel A-D depicts quantification of a different operator's performance. Operator 1 returns the furthest value from the Ground Truth aggregate produced by the other operators (dashed line, panel A), whereas Operator 2 returns the closest value (B). Performance of Operator 3 and 4 is depicted in panels C & D respectively. An analogous approach was used to quantify DeepSlice performance.

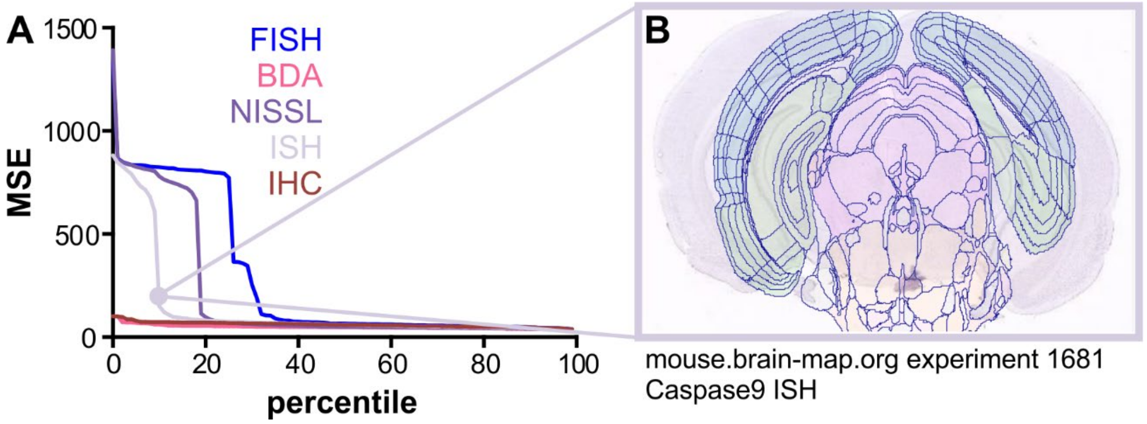

Supplemental Figure 3: Distance between prototype DeepSlice alignments and original alignment metadata

Divergence of prototype DeepSlice predictions from Allen-alignment metadata in FISH, BDA, Nissl, ISH and IHC datasets (A), which were due to errors in the source data. An example of a poorly aligned Allen experiment is shown in B. Loss, measured as mean squared error (MSE) in the prototype model, was used to filter likely spurious experiments from the final training dataset. Abbreviations: FISH: fluorescence in situ hybridization; BDA: biotinylated dextran amine; ISH: in situ hybridization; IHC: immunohistochemistry.

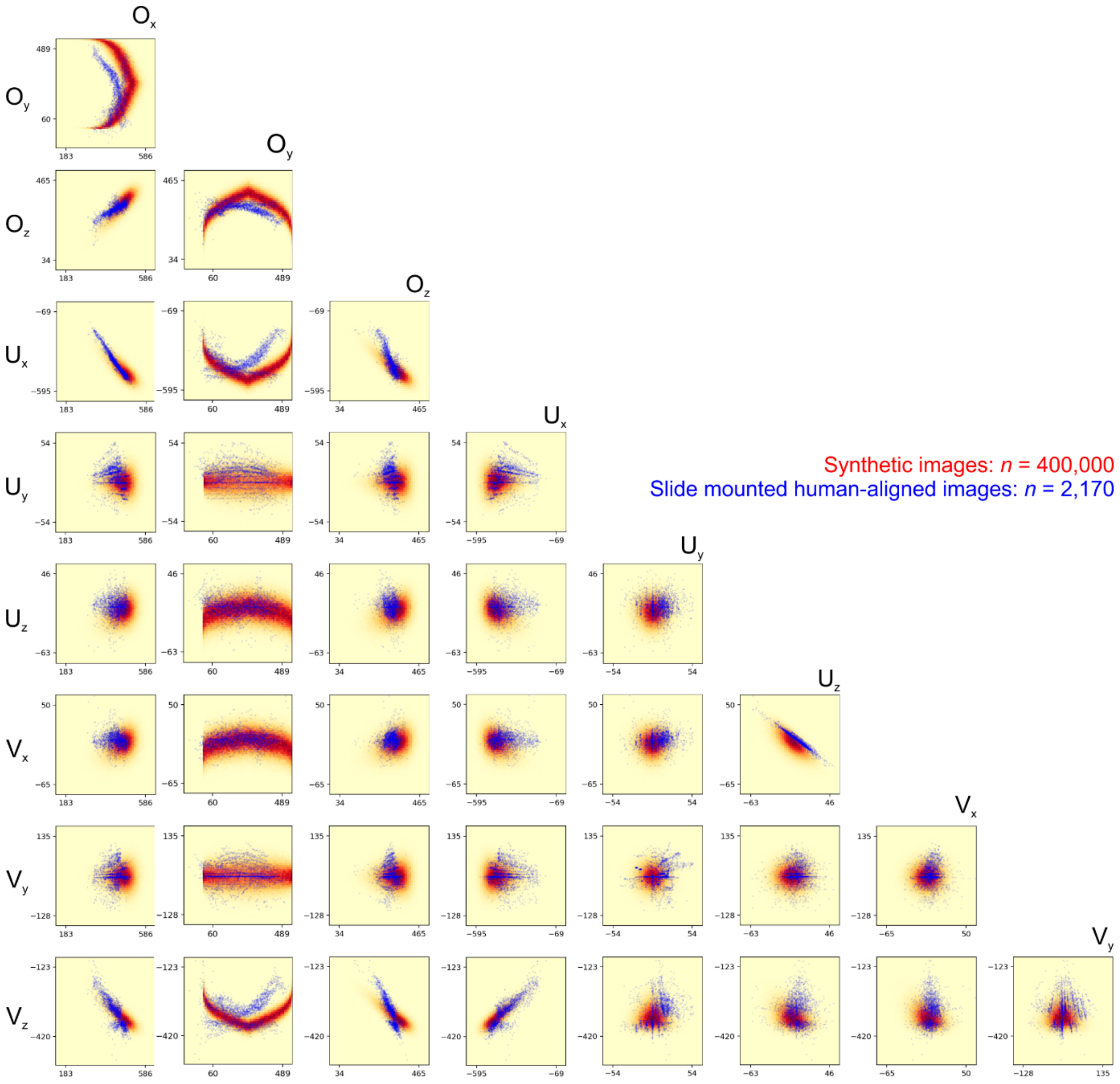

Supplementary Figure 4: The distribution of and relationship between alignment parameters in real and synthetic datasets.

The ranges of and interactions between alignment vectors used to generate 400k synthetic histological images (red) corresponded closely with those assigned by 7 human operators during manual alignment of 305 slide-mounted sections (blue).

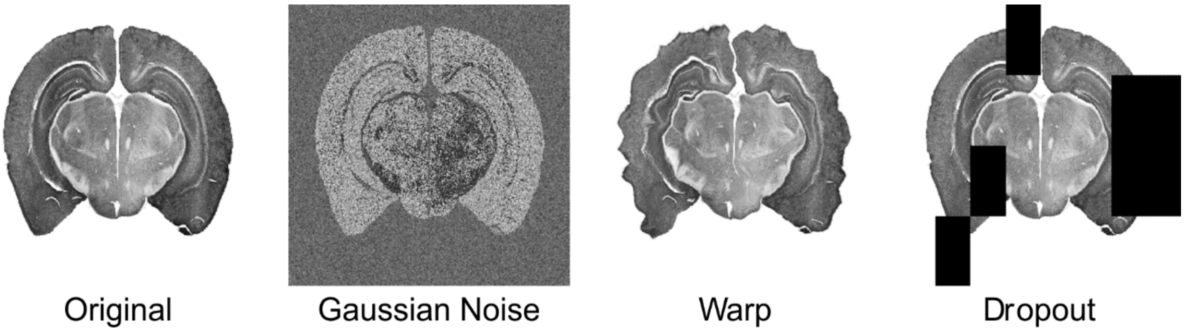

Supplementary Figure 5: Examples of training data preprocessing.

Variability of training data was enhanced by application of filters that randomly assigned combinations of noise, non-linear distortion, and omission of parts of the image. The examples shown illustrate the maximum levels that could be assigned for each filter.

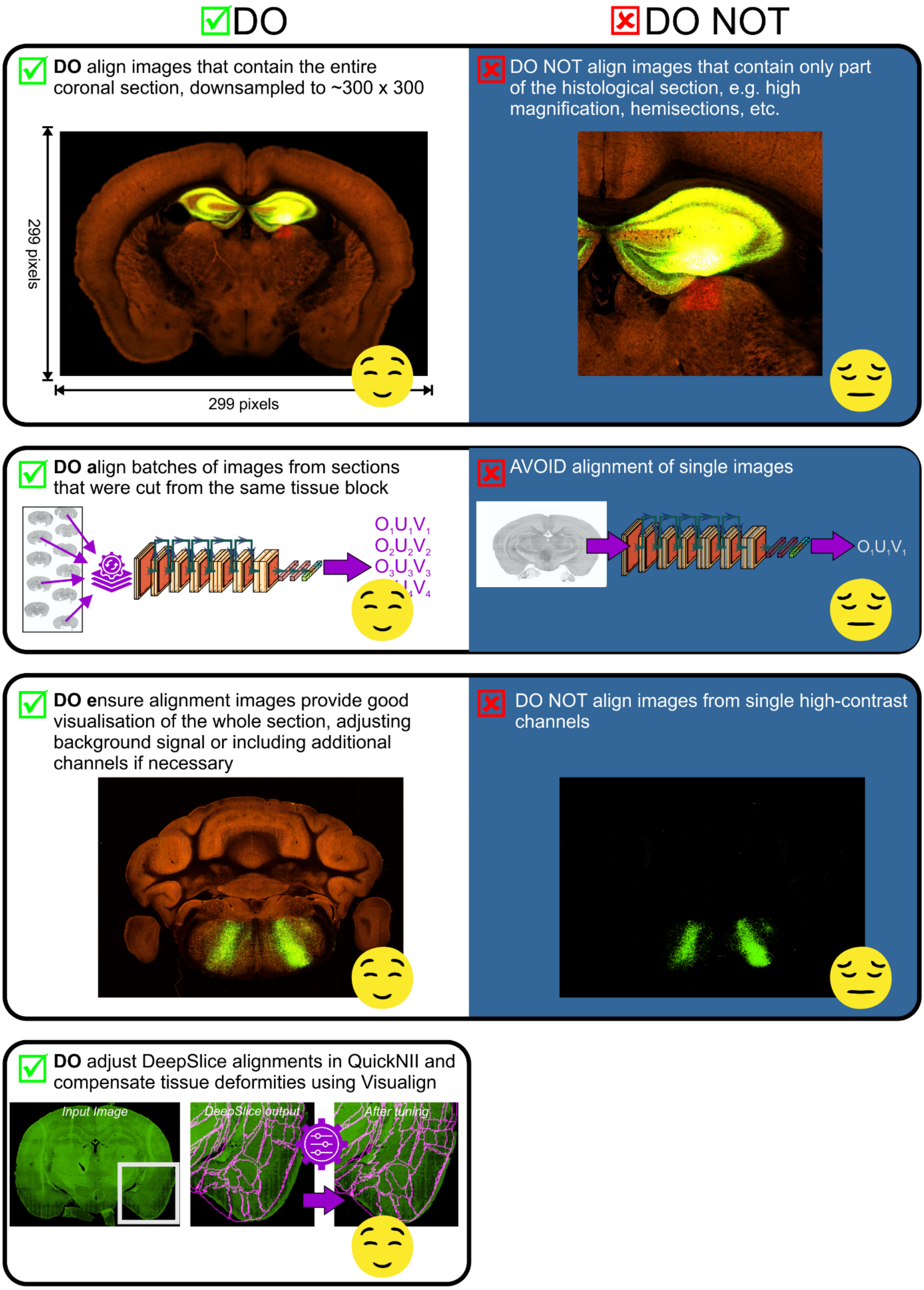

Supplementary Figure 6: Tips and tricks for optimal DeepSlice results.

Infographic highlighting some considerations for optimal DeepSlice use. S2P images courtesy of connectivity.brain-map.org experiments 301122846 & 127041126)[16]. The batch

processing and optimization icons are by Juicy Fish and WARHAMMER, respectively, from Noun Project, and are reproduced under CC BY 3.0 license.
