# Supplementary material for: DeepSlice: rapid fully automatic registration of mouse brain imaging to a volumetric atlas
Source: Nat Commun. 2023 Sep 21;14:5884. doi: 10.1038/s41467-023-41645-4 (PMC10514056; doi:10.1038/s41467-023-41645-4)
Supplement: Supplementary file 3 — Reporting Summary [file 41467_2023_41645_MOESM3_ESM.pdf]

nature portfolio

Corresponding author(s): Simon McMullan

Last updated by author(s): 2023/08/05

Reporting Summary

Nature Portfolio wishes to improve the reproducibility of the work that we publish. This form provides structure for consistency and transparency in reporting. For further information on Nature Portfolio policies, see our Editorial Policies and the Editorial Policy Checklist.

Statistics

For all statistical analyses, confirm that the following items are present in the figure legend, table legend, main text, or Methods section.

n/a	Confirmed	
☐	☒	The exact sample size (*n*) for each experimental group/condition, given as a discrete number and unit of measurement
☒	☐	A statement on whether measurements were taken from distinct samples or whether the same sample was measured repeatedly
☐	☒	The statistical test(s) used AND whether they are one- or two-sided<br>*Only common tests should be described solely by name; describe more complex techniques in the Methods section.*
☒	☐	A description of all covariates tested
☒	☐	A description of any assumptions or corrections, such as tests of normality and adjustment for multiple comparisons
☐	☒	A full description of the statistical parameters including central tendency (e.g. means) or other basic estimates (e.g. regression coefficient) AND variation (e.g. standard deviation) or associated estimates of uncertainty (e.g. confidence intervals)
☐	☒	For null hypothesis testing, the test statistic (e.g. *F*, *t*, *r*) with confidence intervals, effect sizes, degrees of freedom and *P* value noted<br>*Give P values as exact values whenever suitable.*
☒	☐	For Bayesian analysis, information on the choice of priors and Markov chain Monte Carlo settings
☒	☐	For hierarchical and complex designs, identification of the appropriate level for tests and full reporting of outcomes
☒	☐	Estimates of effect sizes (e.g. Cohen's *d*, Pearson's *r*), indicating how they were calculated

Our web collection on statistics for biologists contains articles on many of the points above.

Software and code

Policy information about availability of computer code

Data collection	Analysed data were collated from QuickNII alignment files (human-aligned data), equivalent metadata was downloaded from the Allen Brain Atlas, or produced by the DeepSlice algorithm. Training images were obtained through the Allen Brain Atlas API.
Data analysis	Data were organized using Microsoft Excel or custom code generated using Python 3.7.7. Statistical analysis was performed using Graphpad Prism 9. The average performance of groups of human operators was compared using repeated measures ANOVA (Tukey). Correspondence of cutting angles predicted by DeepSlice with dorsoventral and mediolateral cutting angles contained within S2P image metadata and average human-aligned angle estimates from the Test dataset was quantified by linear regression. P<0.05 was considered statistically significant. Data were plotted using Graphpad Prism 9 or Matplotlib 3.5.3.

For manuscripts utilizing custom algorithms or software that are central to the research but not yet described in published literature, software must be made available to editors and reviewers. We strongly encourage code deposition in a community repository (e.g. GitHub). See the Nature Portfolio guidelines for submitting code & software for further information.

Data

Policy information about availability of data

All manuscripts must include a data availability statement. This statement should provide the following information, where applicable:

- Accession codes, unique identifiers, or web links for publicly available datasets
- A description of any restrictions on data availability
- For clinical datasets or third party data, please ensure that the statement adheres to our policy

Data Availability
Histological images and human alignment data used to generate the Ground Truth library, and alignment predictions generated by the versions of DeepSlice discussed in the current paper, are available in Figshare with the identifier 10.25949/22802411. All other data are available at request from the authors.
Code Availability
The data presented in the current paper were generated using Version 0.3 of DeepSlice, which is available at https://doi.org/10.5281/zenodo.822147160.
The most recent model of the DeepSlice Software package is available on PyPi (https://pypi.org/project/DeepSlice/) and github (10.5281/zenodo.8211292, https://github.com/PolarBean/DeepSlice 61) under an open source license (GPL 3.0). A web application hosting DeepSlice is also available at https://www.deepslice.org.

Research involving human participants, their data, or biological material

Policy information about studies with human participants or human data. See also policy information about sex, gender (identity/presentation), and sexual orientation and race, ethnicity and racism.

Reporting on sex and gender	n/a
Reporting on race, ethnicity, or other socially relevant groupings	n/a
Population characteristics	n/a
Recruitment	n/a
Ethics oversight	n/a

Note that full information on the approval of the study protocol must also be provided in the manuscript.

Field-specific reporting

Please select the one below that is the best fit for your research. If you are not sure, read the appropriate sections before making your selection.

☒ Life sciences ☐ Behavioural & social sciences ☐ Ecological, evolutionary & environmental sciences

For a reference copy of the document with all sections, see nature.com/documents/nr-reporting-summary-flat.pdf

Life sciences study design

All studies must disclose on these points even when the disclosure is negative.

Sample size	No formal sample size estimates were performed. For assessing the accuracy of the DeepSlice algorithm against 2-photon serial block face images, we selected 10 experiments, each consisting of 140 images, at random from the Allen Connectivity Atlas (total atlas database ~443,000 images). Sample-to-sample variability in DeepSlice performance between these 10 experiments was low. For assessment of performance against slide-mounted histological sections that had been independently aligned by a pool of human operators we used data from seven independent biological replicates, consisting of ~300 images, which had been processed using different staining modalities. This sample size was selected based on the availability of data which had been independently aligned by the same pool of human operators.
Data exclusions	Histological images from the caudal brain, which falls outside of the Allen CCF, were excluded from analysis (as they do not map onto the Allen Brain Atlas). A small number of histological images that contained large artifacts (e.g. folded tissue) were also excluded.
Replication	Multiple versions of DeepSlice were generated using the approaches described in this paper. The accuracy of base models varied according to the precise number of training cycles and the speed at which layers were unlocked, but the basic premise of the study, that a CNN can be trained to accurately register mouse brain histology, was upheld in every case.
Randomization	7 slide mounted datasets were divided at random into Validation and Test groups.
Blinding	No blinding of researchers to the identities of human aligners was performed. The researchers did not consider this option at the time.

Reporting for specific materials, systems and methods

We require information from authors about some types of materials, experimental systems and methods used in many studies. Here, indicate whether each material, system or method listed is relevant to your study. If you are not sure if a list item applies to your research, read the appropriate section before selecting a response.

Materials & experimental systems

n/a	Involved in the study
☒	☐ Antibodies
☒	☐ Eukaryotic cell lines
☒	☐ Palaeontology and archaeology
☒	☐ Animals and other organisms
☒	☐ Clinical data
☒	☐ Dual use research of concern
☒	☐ Plants

Methods

n/a	Involved in the study
☒	☐ ChIP-seq
☒	☐ Flow cytometry
☒	☐ MRI-based neuroimaging
